# The journey is just as important as the destination—Digital neuropsychological assessment provides performance stability measures in patients with acquired brain injury

**DOI:** 10.1371/journal.pone.0249886

**Published:** 2021-07-09

**Authors:** Lauriane A. Spreij, Isabel K. Gosselt, Johanna M. A. Visser-Meily, Alex J. Hoogerbrugge, Timo M. Kootstra, Tanja C. W. Nijboer

**Affiliations:** 1 Center of Excellence for Rehabilitation Medicine, UMC Brain Center, University Medical Center Utrecht, Utrecht University and De Hoogstraat Rehabilitation, Utrecht, The Netherlands; 2 Department of Rehabilitation, Physical Therapy Science & Sports, UMC Brain Center, University Medical Center Utrecht, Utrecht University, Utrecht, The Netherlands; 3 Department of Experimental Psychology, Helmholtz Institute, Utrecht University, Utrecht, The Netherlands; Universita degli Studi della Campania Luigi Vanvitelli, ITALY

## Abstract

**Background:**

Cognitive performances on neuropsychological paper-and-pencil tests are generally evaluated quantitatively by examining a final score (e.g., total duration). Digital tests allow for a quantitative evaluation of “how” a patient attained a final score, which opens the possibility to assess more subtle cognitive impairment even when final scores are evaluated as normal. We assessed performance stability (i.e., the number of fluctuations in test performance) to investigate (1) differences in performance stability between patients with acquired brain injury (ABI) and healthy controls; (2) the added value of performance stability measures in patients with ABI; and (3) the relation between performance stability and cognitive complaints in daily life in patients with ABI.

**Methods:**

We administered three digital neuropsychological tests (Rey Auditory Verbal Learning Test, Trail Making Test, Stroop Colour and Word Test) and the Cognitive Complaints—Participation (CoCo-P) inventory in patients with ABI (*n* = 161) and healthy controls (*n* = 91).

**Results:**

Patients with ABI fluctuated more in their performance on all tests, when compared to healthy controls. Furthermore, 4–15% of patients who performed inside normal range on the conventional final scores were outside normal range on the performance stability measures. The performance stability measures, nor the conventional final scores, were associated with cognitive complaints in daily life.

**Conclusions:**

Stability in test performance of patients was clearly dissociable from healthy controls, and may assess additional cognitive weaknesses which might not be observed or objectified with paper-and-pencil tests. More research is needed for developing measures better associated with cognitive complaints.

## Introduction

Neuropsychological paper-and-pencil tests are widely used to assess cognitive impairment [1–4]. Performances on these tests are usually scored by examining a final score, such as the total duration, number of correct responses, or a final drawing [5]. A well-known issue in neuropsychological assessment is the discrepancy between “normal” final scores and the difficulties patients encounter in daily life [6, 7]. An important turning point in neuropsychological assessment was the development of what is now referred to as the "Boston Process Approach" [8]. This method focuses by close observation, on the qualitative analyses of errors and the “process” or the means by which a patient attains a final outcome. Capturing and evaluating the process opens the possibility to assess more subtle cognitive impairment even when final scores are evaluated as normal [5, 9, 10].

A major concern of qualitative analyses is on its inter-rater reliability as differences in outcome may occur between administrators. In addition, determining an abnormal performance (i.e., outside normal range based on the average performance of healthy controls) remains challenging based on qualitative analyses. Digital tests allow a highly detailed registration of data, which enables the development of *quantitative* measures of “how” a patient attained a final outcome. In this study, we capitalized the opportunities afforded by digital tests and developed novel outcome measures to assess more subtle cognitive impairment. We assessed *performance stability* by using three digital neuropsychological tests, namely the Rey Auditory Verbal Learning Test (RAVLT), Trail Making Test (TMT) and the Stroop Colour and Word Test (Stroop). Performance stability is defined as the number of fluctuations in pace (e.g., naming speed in the RAVLT, drawing speed in the TMT). Individuals may show a low stability in test performance, when they respond in an inconsistent pace, outside the normal range based on healthy controls. A low stability in test performance might suggest that underlying processes, such as fluctuating attention or cognitive effort, affect cognitive function negatively [11]. We hypothesized that we would find differences in performance stability between patients with acquired brain injury (ABI) and healthy controls, and that performance stability measures would be of added value in detecting (subtle) cognitive impairment in reference to conventional final scores. Furthermore, neuropsychological tests are often regarded not to be sensitive enough to detect (mild) cognitive difficulties that occur in daily life situations [7, 12–14]. In this study, we describe a first attempt to develop more sensitive measures that might better correspond to difficulties patients encounter in daily life. We explored whether a low stability in test performance would correspond to reported cognitive complaints during daily life activities.

To summarize, we investigated (1) differences in performance stability between patients with acquired brain injury (ABI) and healthy controls; (2) the added value of performance stability measures for patients with ABI only, in reference to conventional final scores; and (3) the relation between performance stability and cognitive complaints in daily life for patients with ABI.

## Materials and methods

### Participants

Participants in this study derived from separate studies in which a digital neuropsychological assessment (d-NPA) was administered. A subset of participants was also part of another study investigating a novel questionnaire assessing cognitive complaints in daily life [15]. All participants gave written informed consent. The experiments were performed in accordance with the Declaration of Helsinki. The research protocols were approved by the Medical Ethics Committee of University Medical Centre Utrecht (METC protocol numbers 16-760/C, 17-407/C, 19-112/C).

We recruited patients with ABI based on the following inclusion criteria: (1) clinically diagnosed with stroke or brain tumour as indicated by clinical computed tomography (CT) or magnetic resonance imaging (MRI) scan, or clinically diagnosed with traumatic brain injury as indicated by a neurologist; (2) aged ≥18 years; (3) fluent in Dutch; (4) patients lived at home at the time of participation; (5) no conventional NPA for clinical purposes in the coming or past three months. Patients were directly invited by clinicians or via an information brochure that was sent by post. The information brochure was also shared with patient associations and on social media. For patients who were willing to participate an appointment was scheduled at the Department of Rehabilitation at *University Medical Centre Utrecht*, *De Hoogstraat Rehabilitation Centre*, or at a patient’s home.

We recruited healthy controls based on the following inclusion criteria: (1) no medical history of neurological and/or psychiatric disorders for which medical treatment was necessary; (2) aged ≥18 years; and (3) fluent in Dutch. Healthy controls were recruited among colleagues and acquaintances, or via an information brochure shared with (sport) associations, or on social media.

### Digital Neuropsychological Assessment (d-NPA)

#### Materials

The d-NPA was administered by a neuropsychologist so no behavioural observations would be lost. The d-NPA contained twelve digital tests which were administered in a fixed order [16]. Attention, memory and executive functioning are generally accepted to be the most basic cognitive functions required to complete tasks and solve everyday problems [6, 15]. As a proof of principle, we started with three tests to cover those basic functions (i.e., RAVLT, TMT, Stroop). We aimed to adopt a similar approach to compute performance stability for each test (see “Measures of performance stability”). The software of the d-NPA was a research prototype created by Philips Research [17]. A tablet was placed in front of the participant and the neuropsychologist sat across them while controlling the tests on a regular laptop. The tablet (Apple© iPad Pro) had a screen size of 12.9-inch and a screen resolution of 2732 × 2048 pixels, and participants used a pencil stylus (Apple© Pencil) which functioned as an ordinary ballpoint pen.

#### Digital tests and conventional outcome measures

*Rey Auditory Verbal Learning Test (RAVLT)*. Participants were required to recall as many words as possible from a list of fifteen words [18]. The words were played on the laptop (volume was set on 100%). This procedure was repeated five times (immediate recall). Subsequently, participants were required to recall the words after 10–20 minutes (delayed recall). The correctly recalled words were used as conventional final scores (immediate recall [0–75] and delayed recall [0–15]).

*Trail Making Test (TMT)*. Participants were required to ’connect-the-dots’ of 25 consecutive targets with the pencil stylus on the tablet [19]. There were two parts to the task: (a) all targets were numbers (1,2,3, etc.) and participants were required to connect them in a sequential order; and (b) targets were numbers and letters and patients were required to alternate between numbers and letters (1, A, 2, B, etc.). Time of completion for both parts separately were used as conventional final score.

*Stroop Colour and Word Test (Stroop)*. In three conditions, items (colour blocks, colour words in black ink, colour words in colour ink) were arranged in a matrix of 10×10 columns and rows and presented on the tablet. Stimuli were presented based on the following: (1) all colours occur an equal number of times, (2) adjacent colours are never the same (so no red-red), (3) all colours appear in each row, (4) the sequence is different for each card [20]. Participants were required to (1) name the colour of the blocks; (2) read the colour word; and (3) name the colour of the ink as fast as possible. Participants were not interrupted. The time of completion per condition was used as conventional final score.

#### Measures of performance stability

The timing of each response was captured, due to an automatic time-stamped data collection. Manual responses (TMT) derived from pen strokes on the tablet screen and were composed in time-stamped coordinates. Pen strokes were classified by being within or outside a target (i.e., circle). Verbal responses (RAVLT, Stroop) were time-based logged by a neuropsychologist by typing the response during the test administration.

**Fig 1:**
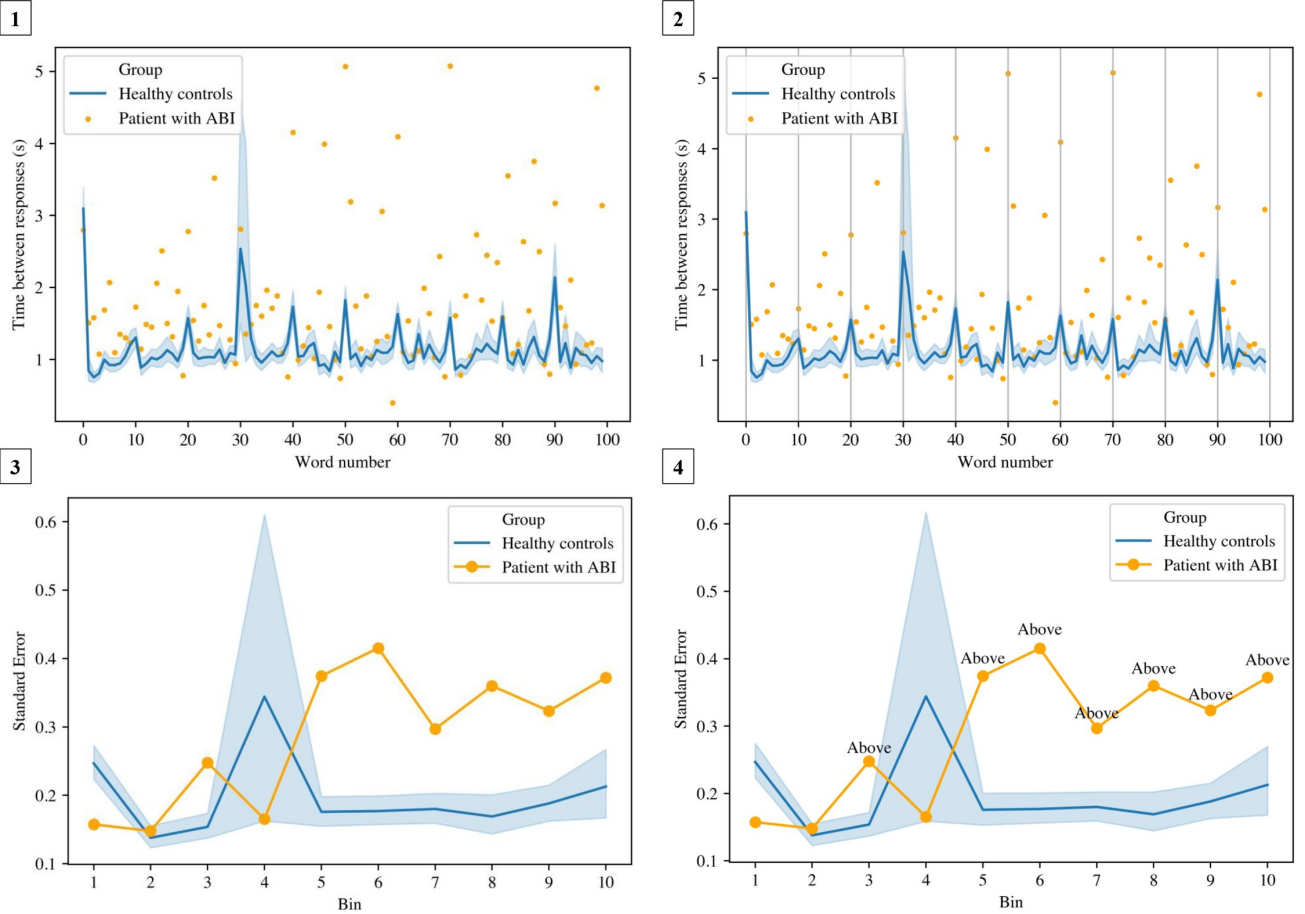
The development of the performance stability measures. The development (in this example the Stroop, condition 3) is illustrated for one patient in four steps: **(1)** The “time between responses” (in seconds) is depicted on the vertical axis, with the orange dots reflecting the time between responses for one patient. The words (100 words in total) are depicted on the horizontal axis. The blue line is the average time of the healthy controls (*n* = 91) with a 95%CI (light blue shade); **(2)** In the Stroop, we computed 10 time bins of 10 words each. The standard error was calculated per time bin; **(3)** Here, the 10 time bins are depicted on the horizontal axis, and the standard error on the vertical axis. The orange line represents the standard error per time bin for one patient. **(4)** The number of time bins in which the standard error of a patient fell above normal range (e.g., 7 out of 10 bins) was computed into a proportion score (e.g., .7).

In the data pre-processing stage, raw files were read and processed with Python 3.7 [21]. See S1 Table for a detailed explanation of the development of performance stability measures and the documentation of missing data analyses. In short, a similar approach was adopted for all three tests (RAVLT, TMT and Stroop) to compute performance stability. First, we determined a time-based measure specific for each test (e.g., time between responses for the Stroop, as indication of “naming speed”). Second, we defined the number of time bins for each test condition (e.g., 10 time bins of 10 words in the Stroop [100 words in total]). The standard error–as measure of variability–was calculated per time bin. We defined a normal range as a 95% Confidence Interval (95%CI) based on the standard errors of the healthy controls, by using the arithmetic mean and standard error of the mean. Next, the standard error was calculated per time bin for each individual patient, and was categorized as below, above or within the normal range of standard errors found in healthy controls. Finally, the number of time bins in which the standard error of a patient fell above normal range (e.g., 7 out of 10 bins) was computed this into a proportion score (e.g., .7). This score reflected *performance stability* (range 0–1), with a higher score indicating a higher number of fluctuations in test performance. See Fig 1 for a visualization of the development of the performance stability measures.

### Cognitive Complaints–Participation (CoCo-P)

Participants were instructed to fill-out the CoCo-P at home and return them by post. The CoCo-P is a patient-reported measure to assess cognitive complaints during daily life activities [15]. The CoCo-P contains 38 items focusing on memory (i.e. retrospective memory, prospective memory), attention (i.e. arousal, orienting, monitoring, sustained) or executive function (i.e. planning, self-evaluating, initiative, mental flexibility) divided over 10 daily life activities (i.e., work/education, leisure activities, travel, driving, finances, use of medication, family life, contact with family, friends and community, cooking, grocery shopping). The response options reflect different grades of independence and effort (0 [independently without effort], 1 [independently with effort], 2 [with help], 3 [not possible], 4 [not applicable]). We computed a *complaint score* per cognitive domain with the following formula: *complaints score* = $\frac{mean\;score}{3}\times 100$. Only items that were applicable for the participant were included. Higher scores indicated a higher degree of reported complaints.

### Demographic and clinical characteristics

We collected data on sex, age and level of education. Level of education was assessed by using a Dutch classification system [22], that consists of seven ranks, with 1 being the lowest (less than primary school) and 7 being the highest (academic degree). These levels were converted into three categories for analysis: low (Verhage 1–4), average (Verhage 5), and high (Verhage 6–7). The Mini-Mental State Examination– 2^nd^ edition (MMSE-2) was administered as indication of general cognitive functioning [23]. In addition, we extracted the following clinical characteristics from the medical files: ABI type, time post ABI onset, and lesion side.

### Statistical analysis

#### Demographic and clinical characteristics

Non-parametric tests (Mann-Whitney *U* test for continuous variables and Chi-square test for categorical variables) were used to compare demographic and clinical characteristics between patients with ABI and healthy controls.

#### Differences in performance stability between patients with ABI and healthy controls

We first presented the results on group level by comparing the *standard error* per stage, time bin, and group for each determined outcome measure per test (RAVLT [in time between responses], Stroop [time between responses], and TMT [time spent within target; drawing speed]). A repeated measures analysis of variance (ANOVA) was performed with stage and time bin (number of stages and time bins varied per test; see S1 Table) as within-subjects variables, and with group (patients with ABI versus healthy controls) as between-subjects variable. We reported the partial eta-squared (*η2*) as effect size, with >.01 reflecting a small effect, >.06, a medium effect, and >.14 a large effect (Cohen, Miles, & Shevlin, 2001) [24].

Several assumptions were evaluated as followed: (1) the distribution of the dependent variable (standard error per stage and time bin) in the two groups was measured with a Shapiro-Wilk normality test. Wherever normality was violated, we evaluated whether outliers influenced the overall results by using Cook’s distances. If there were no influential outliers, no transformation was computed as repeated measure ANOVA is claimed to be more robust to violations of assumptions of normality; (2) sphericity—variances of the differences between all combination—was measured with a Mauchly’s Test. Wherever sphericity was violated, a Greenhouse-Geisser correction was applied.

#### Added value of performance stability measures in reference to conventional final scores

For each test, we calculated the added value of performance stability measures, in reference to the conventional final scores, by determining the percentage of patients who performed inside normal range based on the final scores, but outside normal range based on the performance stability measures. For the performance stability measures, we determined a cut-off based on 2 standard deviations above the average score of healthy controls. Since conventional norms that exist for paper-and-pencil tests cannot be applied on digital versions of the tests [13, 16, 25, 26], we computed percentiles based on healthy controls for each conventional final score to determine a cut-off. A score below 5^th^ percentile was indicative as abnormal performance (see S1 Table for the cut-off scores).

#### Relation between final scores, performance stability and cognitive complaints in daily life

Within patients with ABI only, we computed non-parametric spearman correlations the performance stability measures and the complaints score, and the conventional final scores and the complaints score. An *r* of .1 was considered a small, .3 a moderate, and .5 a large relation [27]. A Benjamini-Hochberg correction was applied, which is considered the best approach in exploratory research [28, 29]. The false discovery rate was set at .1.

## Results

### Demographic and clinical characteristics

We included 160 patients with ABI and 91 healthy controls. See Table 1 for demographical and clinical characteristics per group. There was a comparable amount of men and women in both groups (χ^2^(1, *n* = 252) = 1.76, *p* = .185). Healthy controls were younger than patients with ABI (*U* = 6028.00, *z* = -2.34, *p* = .020) and higher educated (χ^2^(2, *n* = 252) = 6.41, *p* = .041). We investigated the effect of age and education on performance stability within patients and healthy controls (adjusted *p* for 18 tests < .003). There was no significant association between age, education and performance stability on our three digital tests, except for one: the older the healthy controls, the lower the stability in time spent within the target on the TMT A (see S2 Table). Furthermore, patients with ABI scored significantly lower on the MMSE-2 than the healthy controls (*U* = 5346.50, *z* = -3.38, *p* = .001). However, only two patients scored below the cut-off of 24, which indicates that our patient sample was only mild cognitively impaired.

**Table 1:** Demographical and clinical characteristics, split for patients and healthy controls.

	Patients with ABI *n* = 161	*n*	Healthy controls *n* = 91	*n*
**Male (%)**	51.6	*161*	42.9	*91*
**Age in years (mean, SD)**	50.81 (14.47)	*161*	45.68 (17.02)	*91*
**Level of Education (%)**		*161*		*91*
Low	8.7		4.4	
Average	23.6		13.2	
High	67.7		82.4	
**MMSE-2 (0–30) (mean, SD)**	28.38 (1.71)	*158*	29.03 (1.33)	*90*
Below cut-off of 24 (%)	1.2		1.1	
Range	19–30		23–30	
**Time ABI onset (median, range)**	2y (4m – 32y; 2m)	*160*		
**ABI type (%)**		*161*		
Stroke	49.1			
Traumatic Brain Injury	48.4			
Brain tumour (resection)	2.5			
**Lesion side (%)**		*161*		
Left	20.5			
Right	18			
Bilateral	8.7			
Not visible on scan	8.7			
No scan available	44.1			
**Complaints scores (0–100) (mean, SD)**	27.78 (16.91)	*68*	3.58 (6.10)	*33*
**Conventional final scores (mean, SD)**				
RAVLT immediate recall: Recalled words (0–75)	40.62 (13.10)	*158*	47.96 (11.33)	*91*
RAVLT delayed recall: Recalled words (0–15)	8.56 (3.65)	*158*	10.57 (2.97)	*91*
TMT A: Completion time (seconds)	43.02 (19.37)	*159*	32.78 (11.37)	*91*
TMT B: Completion time (seconds)	89.42 (48.97)	*159*	64.02 (25.95)	*91*
Stroop 1: Completion time (seconds)	58.24 (18.91)	*151*	48.06 (8.80)	*89*
Stroop 2: Completion time (seconds)	82.90 (18.51)	*150*	69.64 (11.72)	*87*
Stroop 3: Completion time (seconds)	138.48 (43.37)	*147*	113.36 (25.14)	*87*
**Performance stability measures (0–1) (mean, SD)**				
RAVLT immediate recall: time between responses	.38 (0.23)	*116*	.30 (0.20)	*72*
RAVLT delayed recall: time between responses	.39 (0.34)	*147*	.27 (0.31)	*86*
TMT A: drawing speed	.25 (0.28)	*69*	.31 (0.25)	*48*
TMT B: drawing speed	.17 (0.23)	*69*	.24 (0.30)	*48*
TMT A: time within target	.31 (0.24)	*54*	.23 (0.24)	*44*
TMT B: time within target	.26 (0.23)	*54*	.21 (0.24)	*44*
Stroop 1: time between responses	.55 (0.30)	*142*	.29 (0.24)	*82*
Stroop 2: time between responses	.44 (0.27)	*142*	.24 (0.22)	*82*
Stroop 3: time between responses	.39 (0.27)	*142*	.24 (0.20)	*82*

**Abbreviations**: (ABI) Acquired Brain Injury; years (y); months (m); Standard Deviation (SD), Mini-Mental State Examination– 2^nd^ version (MMSE-2); Rey Auditory Verbal Learning Test (RAVLT), (SD) Standard Deviation; Trail Making Test (TMT); Stroop Color and Word Test (Stroop). Note. A higher complaints score is indicative for a higher degree of reported complaints. Higher performance stability scores are indicative for a higher number of fluctuations in test performance.

### Differences in performance stability between patients with ABI and healthy controls

#### Rey auditory Verbal Learning Test (RAVLT)–immediate and delayed recall

Regarding the immediate recall, patients with ABI fluctuated more in naming speed (with time between responses as outcome measure) than healthy controls (*F (*1, 186) = 5.00, *p* = .027, *η2* = .026). All participants fluctuated more in naming speed in the first trial compared to the following four trials (*F* (3.68, 744) = 4.97, *p* = .001, *η2* = .026), and more in the second half of a trial compared to the first half of a trial (*F* (1, 186) = 132.40, *p* < .001, *η2* = .416). There were no interaction effects.

**Fig 2:**
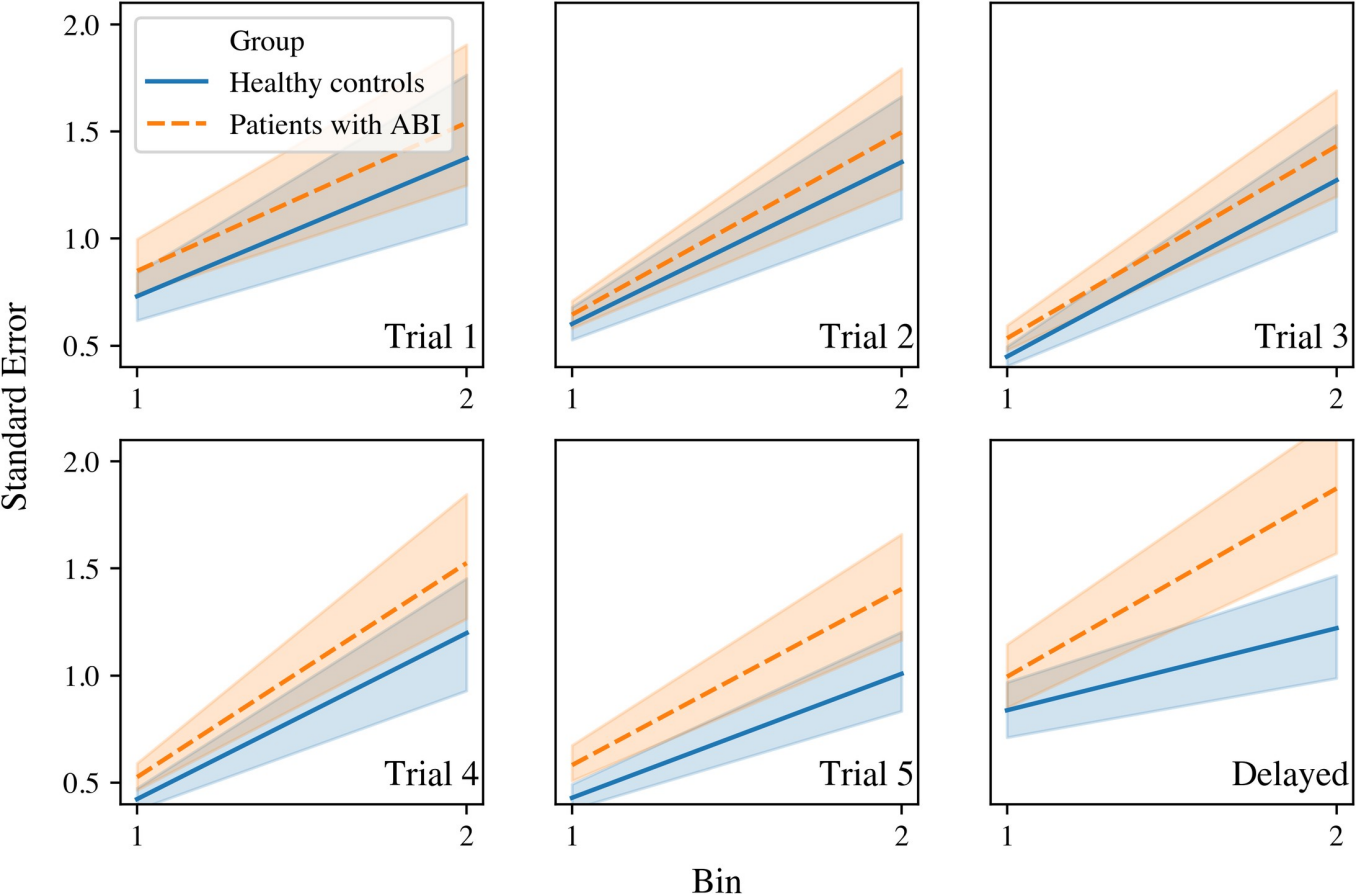
Performance stability in naming speed (with time between responses as outcome measure) on the RAVLT immediate recall (trial 1, 2, 3, 4 and 5) and delayed recall at group level. Each trial was divided into 2 bins (horizontal axis). The standard error (as measure of variability) is depicted on the vertical axis. Patients fluctuated significantly more in naming speed compared to healthy controls.

Regarding the delayed recall, patients with ABI fluctuated more in naming speed (time between responses) than healthy controls (*F* (1, 231) = 342.99, *p* = .003, *η2* = .038). Additionally, all participants fluctuated more in naming speed in the second half of the test compared to the first half ((*F* (1, 231) = 23.83, *p* < .001, *η2* = .093). There was no interaction effect. See Fig 2 for a visualization of the effects.

#### Trail Making Test (TMT)–part A and B

Patients with ABI did not fluctuate more in drawing speed than healthy controls (*F* (1,115) = 2.47, *p* = .119, *η2* = .021). All participants fluctuated more in drawing speed in part B compared to part A (*F* (1, 115) = 22.93, *p* < .001, *η2* = .166). Additionally, participants fluctuated more in drawing speed in the first bin compared to the second bin, and increasing fluctuations in the last three time bins (*F* (3.73, 460) = 80.99, *p* < .001, *η2* = .413). There was an interaction effect of stage and time bin (*F* (2.97, 460) = 9.15, *p* < .001, *η2* = .074), indicating that part A reflected a different pattern of fluctuations compared to part B.

**Fig 3:**
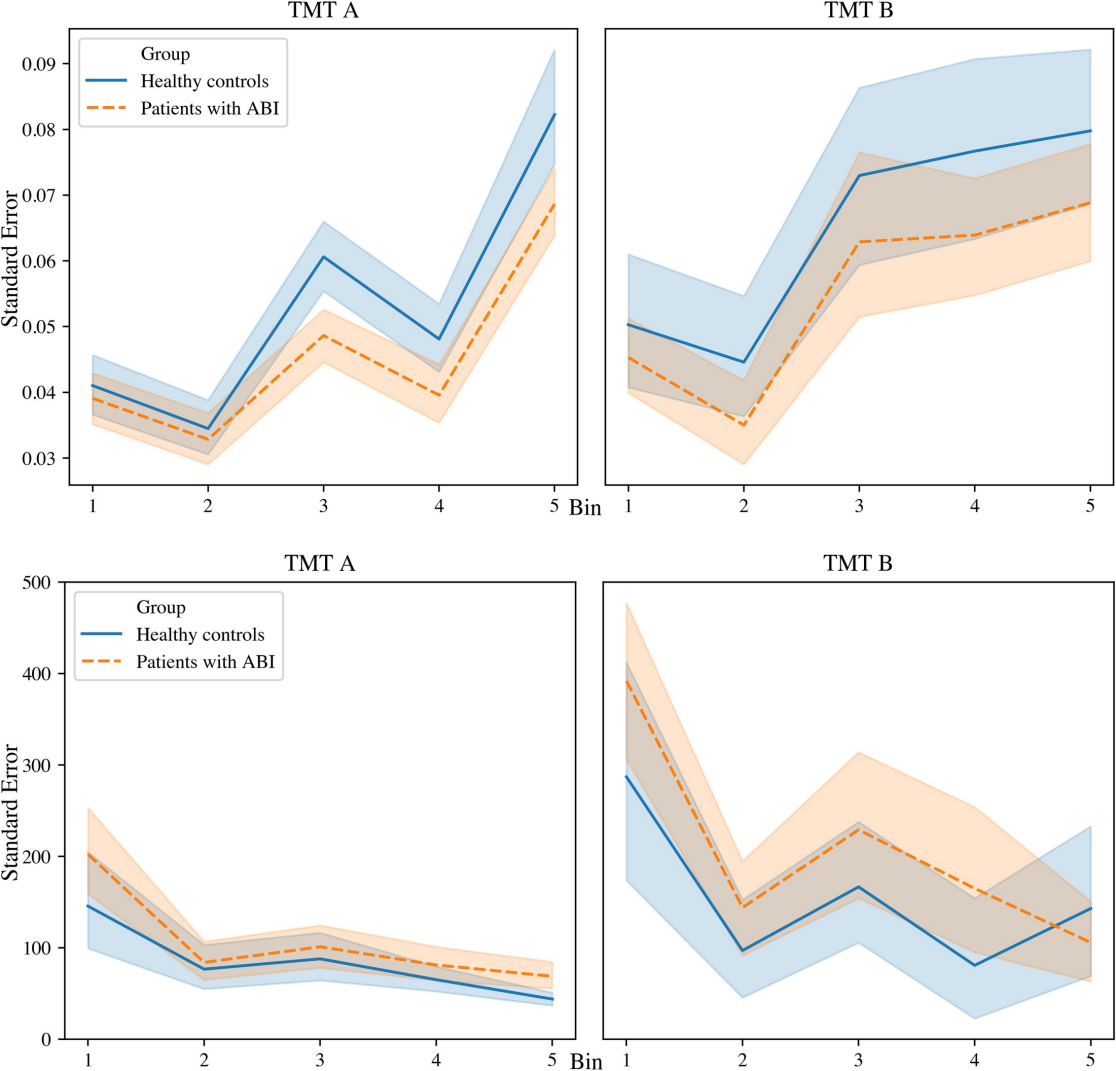
**a. Performance stability in drawing speed on the TMT (part A and B) at group level.** Each stage was divided into 5 bins of 5 targets (horizontal axis). The standard error is depicted on the vertical axis. The performance stability in drawing speed of patients and healthy controls was comparable. **b. Performance stability in “thinking/searching time” (with time spent within a target as outcome measures) on the TMT (part A and B) at group level.** Each stage was divided into 5 bins of 5 targets (horizontal axis). The standard error is depicted on the vertical axis. Patients fluctuated significantly more in “thinking/searching time” compared to healthy controls.

Patients with ABI fluctuated more in “thinking/searching time” (with time spent within a target as outcome measures) than healthy controls (*F* (1, 96) = 4.27, *p* = .042, *η2* = .043). All participants fluctuated more in “thinking/searching time” in part B compared to part A (*F (*1, 96) = 144.34, *p* < .001, *η2* = .601), and more in the first time bin compared to the following four time bins (*F* (2.76, 384) = 21.50, *p* < .001, η2 = .183). There were no interaction effects. See Fig 3A and 3B for a visualization of the effects.

#### Stroop Color and Word Test (Stroop)–condition 1, 2 and 3

**Fig 4:**
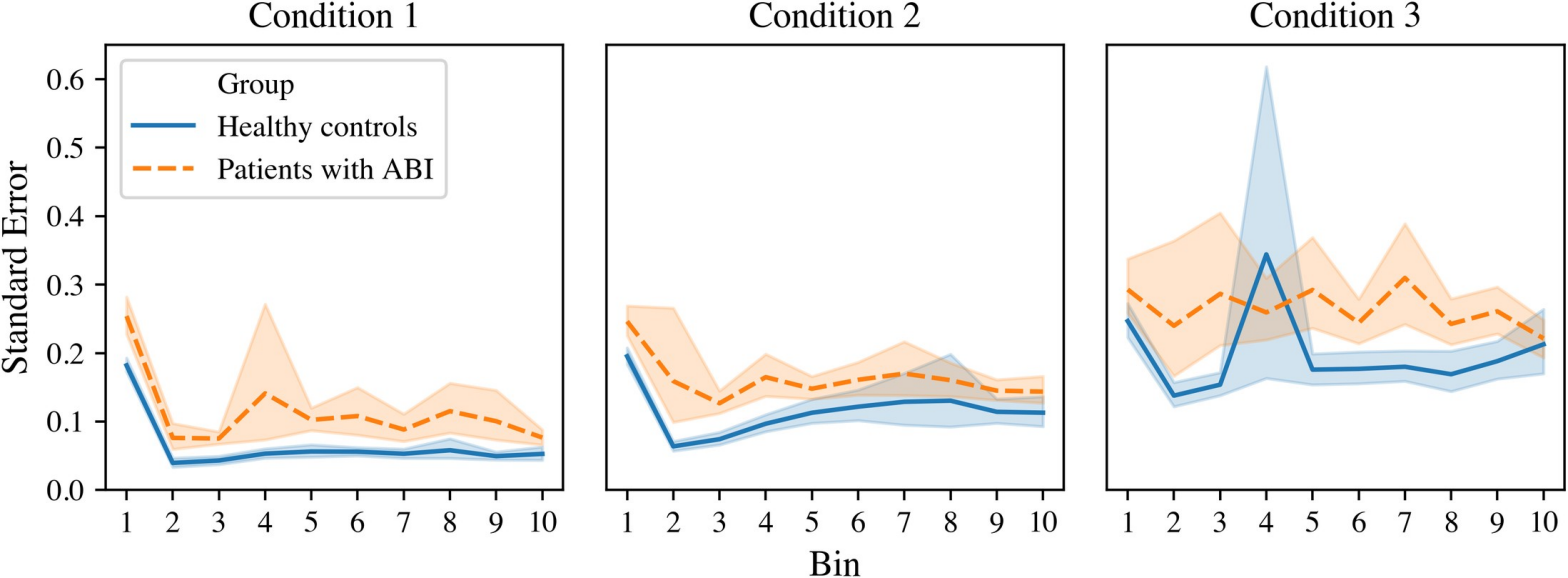
Performance stability in naming speed (with time between responses as outcome measure) on the Stroop (condition 1, 2 and 3) at group level. Each condition was divided into 10 bins of 10 words (horizontal axis). The standard error is depicted on the horizontal axis. Patients fluctuated significantly more in naming speed compared to healthy controls.

Patients with ABI fluctuated more in naming speed (with time between responses as outcome measure) than healthy controls (*F* (1, 222) = 13.83, *p* < .001, *η2* = .059). All participants showed increasing fluctuations throughout the conditions (*F* (1.61, 444) = 64.72, *p* = .001, *η2* = .226). Additionally, all participants fluctuated more in naming speed in the first time bin compared to the following nine time bins (*F* (3.31, 1998) = 5,02, *p* < .001, *η2* = .022). There were no interactions effects. See Fig 4 for a visualization of the effects.

### Added value of performance stability measures in reference to conventional final scores

Overall, 2–12% of patients performed outside normal range on the conventional final scores (defined as <5^th^ percentile based on data of healthy controls). With regard to the added value, 4–15% of patients performed inside normal range on the conventional final scores, but outside normal range on the performance stability measures (Table 2).

**Table 2:** Percentages of patients outside normal range based on performance stability measures in reference to conventional final scores.

	Patients performing outside normal range on final scores (%)	*Added value*: patients who performed inside normal range on final scores, but outside normal range on performance stability measures (%).	*n*
**RAVLT Immediate recall:** total words x stability	6	10.3	*116*
**RAVLT Delayed recall**: total words x stability	10.9	12.2	*147*
**TMT A**: total time x stability (drawing speed)	4.3	5.8	*69*
**TMT B**: total time x stability (drawing speed)	2.9	4.3	*69*
**TMT A**: total time x stability (time within target)	3.7	5.6	*54*
**TMT B**: total time x stability (time within target)	1.9	3.7	*54*
**Stroop 1:** total time x stability	12	14.8	*142*
**Stroop 2:** total time x stability	9.2	4.2	*142*
**Stroop 3:** total time x stability	3.5	10.6	*142*

### Relation between final scores, performance stability and cognitive complaints in daily life

There were no significant relations between the conventional final scores and the subjective cognitive complaints, nor between performance stability and the subjective cognitive complaints (Table 3).

**Table 3:** Relation between conventional final scores, performance stability measures, and reported cognitive complaints, within patients with ABI only.

	Cognitive Complaints Score	
**RAVLT Immediate recall**	***rs (p-value)***	***n***
Conventional final score (total words)	-.002 (*p* = .987)	*50*
Performance stability measure	.15 (*p* = .286)	*50*
**RAVLT Delayed recall**		
Conventional final score (total words)	-.02 (*p* = .892)	*60*
Performance stability measure	.24 (*p* = .069)	*60*
**TMT A Drawing Speed**		
Conventional final score (total time)	.19 (*p* = .301)	*31*
Performance stability measure	-.31 (*p* = .094)	*31*
**TMT B Drawing Speed**		
Conventional final score (total time)	.41 (*p* = .022)	*31*
Performance stability measure	-.44 (*p* = .013)	*31*
**TMT A Time spent within target**		
Conventional final score (total time)	.05 (*p* = .828)	*23*
Performance stability measure	-.20 (*p* = .360)	*23*
**TMT B Time spent within target**		
Conventional final score (total time)	.34 (*p* = .108)	*23*
Performance stability measure	.04 (*p* = .848)	*23*
**Stroop Condition 1**		
Conventional final score (total time)	.30 (*p* = .026)	*56*
Performance stability measure	.16 (*p* = .241)	*56*
**Stroop Condition 2**		
Conventional final score (total time)	.32 (*p* = .018)	*56*
Performance stability measure	.29 (*p* = .029)	*56*
**Stroop Condition 3**		
Conventional final score (total time)	.25 (*p* = .068)	*56*
Performance stability measure	.17 (*p* = .216)	*56*

*No significant p-values based on a Benjamini-Hochberg correction. Note. Higher complaints score are indicative for a higher degree of reported complaints. Higher performance stability scores are indicative for a higher number of fluctuations in test performance.

## Discussion

In this study, we capitalized the opportunities afforded by digital neuropsychological tests and developed novel outcome measures targeting performance stability to assess more subtle cognitive impairment. We investigated (1) differences in performance stability between patients with acquired brain injury (ABI) and healthy controls; (2) the added value of performance stability measures for patients with ABI only, in reference to conventional final scores; and (3) the relation between performance stability and cognitive complaints in daily life for patients with ABI.

Patients with ABI fluctuated significantly more in naming speed during the RAVLT and Stroop compared to healthy controls, suggesting that patients responded with a less consistent pace. In the TMT, patients with ABI fluctuated more in “thinking/searching time” compared to healthy controls. On all novel outcome measure patients were clearly dissociable from healthy controls, except for the performance stability in drawing speed during the TMT. This indicates that healthy controls and patients showed a comparable number of fluctuations in their drawing speed, probably due to accelerations on certain points in the test (e.g., when consecutive targets are not far apart). Furthermore, 2–12% of patients performed outside normal range on the conventional final scores. When developing novel outcome measures, it is important to investigate whether an additional outcome measure improves the diagnostic accuracy by going beyond the available diagnostic information [30]. The added value involves the 4–15% of patients who performed inside normal range on the conventional final scores, but outside normal range on the performance stability measures. This might be considered as an important clinically relevant finding, as we were able to objectify cognitive impairment among those patients, which would not have been objectified with a paper-and-pencil administration. Finally, the performance stability measures, nor the conventional final scores, were associated with cognitive complaints in daily life.

How can we explain the differences in performance stability between patients with ABI and healthy controls? A low stability in test performance might suggest that underlying process, such as fluctuating cognitive effort, affect cognitive function negatively [11]. Cognitive effort refers to the extent in which an individual exerts an adequate level of effort to execute a cognitive task. For example, during the TMT, participants fluctuated more in the more complex part of the test (part B), when compared to the less complex part (part A). Performances on part B are associated with more complex visual sequencing and inhibitory control, whereas performances on part A are often associated with primarily visual-scanning and psychomotor processing speed [31]. An elevated number of fluctuations in the more complex stage might reflect an elevated level of cognitive effort that is required from patients to execute the task, suggesting that fluctuations in test performance are more likely to occur during more complex tasks. Another process underlying a low stability in test performance, might involve fluctuations in attention. Attention is integral to cognition, and therefore affects performances on almost every cognitive task in daily life. Hence, assessing fluctuations in attention seems crucial in neuropsychological assessment following ABI. Fluctuations in attention, however, are difficult to measure with neuropsychological paper-and-pencil tests and they are often difficult to observe, especially when they are small and/or short in duration. The impact of small and short fluctuations in attention is therefore largely unknown. A starting point comes from functional magnetic resonance imaging (fMRI) studies indicating that the strength of functional brain networks predicts sustained attention [32, 33]. When the integrity of functional or structural brain networks is hampered, this results in a range of cognitive impairments, from short drops in performance [33] up to severe cognitive deficits [34, 35]. As fMRI is not always feasible as care as usual in clinical settings and test-retest reliability in individual differences research has been a concern [36], it is of utmost importance to further develop cognitive (behavioural) measures to identify fluctuations in attention and their effects on behaviour (i.e., test performance). This study was a first attempt to develop such outcome measures targeting fluctuations during test performance.

The performance stability measures, nor the conventional final scores, were associated with cognitive complaints in daily life. This might be explained by the fact that cognitive impairment (as measured with neuropsychological tests) are not necessarily an indication of cognitive complaints, and vice versa [37–40]. Psychological factors (e.g., coping styles, depressive symptoms) and environmental factors (e.g., domestic or vocational modifications) might influence subjective reports, which is likely the reason why cognitive impairment neither predict or explain cognitive complaints very well [41]. Another explanation might be that neuropsychological tests do not correspond to everyday functioning [7, 12]. Neuropsychological tests target cognitive functions in isolation (e.g., verbal memory, planning), whereas daily life tasks require multiple cognitive functions at once. In addition, neuropsychological tests are administered under optimal conditions in a quiet and non-distracting environment to elicit the patient’s best possible performance. Even though digital tests might open the possibility to develop more sensitive outcome measures [13], the setting in which they are administered does still not correspond to daily life. More advanced technologies, such as Virtual Reality, have the potential to assess cognitive impairment in simulated environment resembling daily life [42, 43]. However, this study was only a first attempt to develop more sensitive measures to assess more subtle cognitive impairment. More development and research is needed in this area [13].

### Strengths and limitations

A strength of this study was the inclusion of a large number of patients with ABI (*n* = 161) and the broad recruitment via clinicians, associations and social media, which increases the representativeness of our sample. A general concern might regard a potential selection bias, where patients who are willing to participate are probably patients who are less impaired and more highly educated [44, 45]. Indeed, our sample was relatively mild cognitively impaired (2–12% performed outside normal range on conventional final scores) and more highly educated (68%). For this reason, our findings might not be generalized to a broader sample, which might be considered as a limitation. However, including patients with mild cognitive impairment might be considered as a strength, as developing more sensitive outcome measures is crucial for this group. In addition, it is to be expected that a lower stability in test performance occurs more frequently in patients who are more cognitively impaired, which would have strengthen the results.

We intentionally aimed to include a heterogeneous sample to explore performance stability in patients with ABI. However, one could argue that a heterogeneous sample is a potential limitation, as each brain injury has a different pathology. Injury characteristics were not systematically noted in the medical files, and we were therefore unable to further investigate specific subgroups within our patient sample. For example, it would have been interesting to investigate whether the severity of stroke (e.g., National Institutes of Health Stroke Scale), TBI (e.g., Glasgow Coma Scale, duration loss of consciousness or post-traumatic amnesia) and tumour grade (grade I–V of the World Health Organization) would affect performance stability. Moreover, it might be interesting to investigate the relation of performance stability and the lesion location or the damage to brain networks, by using brain imaging techniques like diffusion tensor imaging (DTI) on group level. In this study, the time post injury varied between 4 months and 32 years, indicating that patients were in different phases post-injury. Future research should include a large sample of patients, which will allow for the exploration of possible differences in performance stability between specific subgroups regarding clinical characteristics (e.g., diagnosis, severity, time post injury).

Furthermore, we only included three tests (RAVLT, TMT, Stroop), for which we adopted a similar approach to compute performance stability. Performance stability measures were not as easily developed for other tests in the test battery. For instance, in drawing tests (i.e., Rey-Osterrieth Complex Figure, Cube drawing, Clock Drawing) computational methods are needed to analyse how drawings are constructed. Although first attempts are described in the literature to evaluate the process of construction in drawing [9, 10, 46], the development of performance stability measures are still ongoing. Furthermore, other tests (i.e., Digit Span, Verbal Fluency) were not appropriate, since time bins were not easily defined without extensive data loss.

Finally, we used the CoCo-P inventory to assess cognitive complaints in daily life. One might argue that the perspective of a significant other (e.g., caregiver, relative) might have had added value to the evaluation of cognitive complaints in daily life of patients with ABI, especially as the over- or underestimation of cognitive abilities is a common issue in patients with ABI [15, 47–49]. For this reason, future research might consider including the perspective of a significant other when investigating cognitive complaints in daily life. This primary aim for the current study was, however, to develop and investigate performance stability measures and explore its relation with self-reported cognitive complaints in daily life.

### Clinical implications

So far, observations of behaviour while performing a test provide important pieces of information regarding performance stability. For instance, neuropsychologists may observe certain behavioural signs that indicate a low stability during a test (i.e., fluctuating between a fast/slow pace, a weakened pace towards the end of the test). Observations, however, might vary significantly among neuropsychologists due to differences in interpretation. Digital tests allow for quantitative measures of performance stability, without interfering with the conventional measures.

### Future research

Previous research reported a significant gap in the application of digital tests to further improve cognitive assessment [13]. This study was just a first step in the development of novel outcome measures assessing performance stability. The "Boston Process Approach" method focusses on the analyses of errors and the process or the means by which a patient reaches a solution to a problem [8, 11, 50]. Although this process approach is developed to be applied on paper-and-pencil tests, recent research has incorporated the approach in several digital tests [5]. In our study, we only focussed on performance stability, but additionally integrating the analysis of errors and detection of behavioural patterns might capitalize the opportunities afforded by digital tests.

Furthermore, future research should investigate the underlying processes that might influence performance stability, such as fluctuating attention or cognitive effort. Different psychophysiological techniques including measures of heart function (e.g., heart rate variability), brain activity (e.g., task-evoked brain potentials), and eye-tracking features (e.g., pupillary dilation, blink rate) have been used to measure cognitive effort, cognitive load, (mental) stress or fatigue [51–53]. For example, the increase or decrease in pupil diameter while processing a cognitive task reflects small differences in cognitive effort. Psychophysiological techniques may provide added value not captured through behavioural or self-report measures alone, and may provide insight into the underlying processes influencing performance stability.

## Conclusions

In this study, we capitalized the opportunities afforded by digital neuropsychological tests and developed novel outcome measures to assess more subtle cognitive impairment. We assessed *performance stability* by evaluating the number of fluctuations in test performance on three digital neuropsychological tests. Patients with ABI showed a higher number of fluctuations in their performance on the RAVLT, TMT and Stroop, when compared to healthy controls. The added value involved the 4–15% of patients who performed inside normal range on the conventional final scores, but outside normal range on the performance stability measures. This study was a first attempt to develop more sensitive measures to assess mild cognitive impairment, which cannot be quantified at this level of (objective) detail with paper-and-pencil tests. More development and research is needed in this area.

## Supporting information

S1 Table Development of measures of cognitive stability. (DOCX)

S2 Table Effect of age and education on performance stability within patients and healthy controls. (DOCX)
