# Supplementary material for: The journey is just as important as the destination—Digital neuropsychological assessment provides performance stability measures in patients with acquired brain injury
Source: PLoS One. 2021 Jul 9;16(7):e0249886. doi: 10.1371/journal.pone.0249886 (PMC8270450; doi:10.1371/journal.pone.0249886)
Supplement: S1 Table — (DOCX) [file pone.0249886.s001.docx]

**S1 Table . Development of measures of cognitive stability.**

|  | Conventional final scores and the cut-off based on the 5^th^ percentiles of health controls. | **Step 1** | **Step 2** | **Step 3** | **Step 4** | **Step 5** | **Data loss** |
| --- | --- | --- | --- | --- | --- | --- | --- |
|  |  | Outcome measures | Time bins | Computing 95% CI - Healthy controls | Computing Standard Error (SE) - Individuals | Computing standardized measures |  |
| **RAVLT immediate recall (Trial 1-5)** | Correct recalled words (0-75); Cut-off: <26 is considered abnormal (based on healthy controls). | Time between consecutive responses^1^ | As the length of a trials differs between participants, we chose 2 bins (total time / 2) per stage = 5 stage x 2 time bins | As measure of variability, the SE and a 95%CI was calculated per time bin based on 72 healthy controls in the immediate condition, and 86 healthy controls in the delayed condition. | The SE was calculated per time bin for each individual patient. The SE could be categorized as below, above or within the 95%CI of healthy controls. | We counted how many times the standard error of a patient fell outside normal range and computed this into a proportion (0-1). | In the immediate stage, we reduced the data loss to 2.8% with linear interpolation, for at most of one consecutive missing timestamp. In the delayed stage, we reduced it to 6.8%. Participants with a missing SE in any time bin (due to >1 consecutive missing timestamps) were excluded from the analyses, resulting in 72 healthy controls and 116 patients in the immediate condition, and 86 healthy controls and 147 patients in the delayed condition. |
| **RAVLT delayed recall** | Correct recalled words (0-15); Cut-off: <5 is considered abnormal. |  |  |  |  |  |  |
| **TMT Part A** | Completion time part A (seconds); Cut-off: >61.28 is considered abnormal. | Time spend within target^2^, drawing speed^3^ | 5 bins (5 x 5 targets) | As measure of variability, the SE and a 95%CI was calculated per time bin based on 48 healthy controls with drawing speed as outcome measure, and 44 healthy controls with time spent within target as outcome measure. | The SE was calculated per time bin for each individual patient. The SE could be categorized as below, above or within the 95%CI of healthy controls. | We counted how many times the standard error of a patient fell outside normal range and computed this into a proportion (0-1). | Participants with a missing SE in any time bin (when there were <2 timestamps) were excluded from the analyses, resulting in 48 healthy controls and 69 patients (with drawing time as outcome measures), and 44 healthy controls and 54 patients (with time spent within target). |
| **TMT Part B** | Completion time part B (seconds); Cut-off: >125.42 is considered abnormal. |  |  |  |  |  |  |
|  |  |  |  |  |  |  |  |
| **Stroop Condition 1** | Completion time (seconds); Cut-off: >59.69 is considered abnormal. | Time between consecutive responses^1^ | 10 bins (10 x 10 items) per stage = 3 stages x 10 time bins | As measure of variability, the SE and a 95%CI was calculated per time bin based on 82 healthy controls. | The SE was calculated per time bin for each individual patient. The SE could be categorized as below, above or within the 95%CI of healthy controls. | We counted how many times the standard error of a patient fell outside normal range and computed this into a proportion (0-1). | A total of 8% of the data was not usable, due to an error in the data files or due to >1 consecutive missing timestamps. Linear interpolation was not possible due to non-linear data. Participants with a missing SE in any time bin were excluded from the analyses, resulting 82 healthy controls and 142 patients. |
| **Stroop Condition 2** | Completion time (seconds); Cut-off: >93.13 is considered abnormal. |  |  |  |  |  |  |
| **Stroop Condition 3** | Completion time (seconds) Cut-off: >163.45 is considered abnormal. |  |  |  |  |  |  |

Note. (1) Time between responses was calculated as $interResponseTime{=responseTimeStamp}_{n}-startTime$, where *n* is the first response. For $n>1$, time between responses was calculated as $interResponseTime= {responseTimeStamp}_{n}- {responseTimeStamp}_{n-1}$. Additionally, the raw datafiles included each response whether it was correct or incorrect; all responses were used regardless of correctness. (2) Time spent within target was calculated as the total amount of time that the participant spent inside a target with the pencil stylus, by subtracting *first timestamp crossing the border to enter the target (starting point)*, from *the first timestamp crossing the border to leave the target (endpoint).* (3) Drawing speed was calculated as the total distance (in pixels) drawing from one target to another target, divided by the time it took a participant to do so.
