# Supplementary material for: The journey is just as important as the destination—Digital neuropsychological assessment provides performance stability measures in patients with acquired brain injury
Source: PLoS One. 2021 Jul 9;16(7):e0249886. doi: 10.1371/journal.pone.0249886 (PMC8270450; doi:10.1371/journal.pone.0249886)
Supplement: S2 Table — (DOCX) [file pone.0249886.s002.docx]

**S2 Table . Effect of age and education on performance stability within patients and healthy controls.**

|  | **Patients with ABI (***n*=161) | | | | **Healthy controls (***n*=91) | | | |
| --- | --- | --- | --- | --- | --- | --- | --- | --- |
|  | *Age* | ***n*** | *Education* | ***n*** | *Age* | ***n*** | *Education* | ***n*** |
| **Performance stability measures (0-1)** |  |  |  |  |  |  |  |  |
| RAVLT immediate recall: time between responses | *rs* = .22, *p*=.021 | *116* | *H*(2)=3.46, *p*=.117 | *116* | *rs* = .24 (*p* = .044) | *72* | *H*(2) = 2.24, *p* = .326 | *72* |
| RAVLT delayed recall: time between responses | *rs*=.04, *p*=.625 | *147* | *H*(2)=4.23, *p*=.121 | *147* | *rs* = .27 (*p* = .011) | *86* | *H*(2) = 0.85, *p* = .654 | *86* |
| TMT A: drawing speed | *rs*=-.18, *p*=.133 | *69* | *H*(2)=4.05, *p*=.132 | *69* | *rs* = -.15 (*p* = .314) | *48* | *H*(2) = 1.95, *p* = .384 | *48* |
| TMT B: drawing speed | *rs*=-.01, *p*=.925 | *69* | *H*(2)=1.45, *p*=.486 | *69* | *rs* = -.06 (*p* = .698) | *48* | *H*(2) = 1.61, *p* = .447 | *48* |
| TMT A: time within target | *rs*=.31, *p*=.023 | *54* | *H*(2)=8.40, *p*=.015 | *54* | *rs* = .55 (*p* < .001)* | *44* | *H*(2) = 2.71, *p* = .258 | *44* |
| TMT B: time within target | *rs*=.27, *p*=.05 | *54* | *H*(2)=6.26, *p*=.044 | *54* | *rs* = .34 (*p* = .023) | *44* | *H*(2) = 2.46, *p* = .293 | *44* |
| Stroop 1: time between responses | *rs*=.14, *p*=.109 | *142* | *H*(2)=3.74, *p*=.154 | *142* | *rs* = .20 (*p* = .067) | *82* | *H*(2) = 3.58, *p* = .167 | *82* |
| Stroop 2: time between responses | *rs*=.18, *p*=.029 | *142* | *H*(2)=2.39, *p*=.303 | *142* | *rs* = .21 (*p* = .063) | *82* | *H*(2) = 5.53, *p* = .063 | *82* |
| Stroop 3: time between responses | *rs*=.11, *p*=.18 | *142* | *H*(2)=1.53, *p*=.465 | *142* | *rs* = .22 (*p* = .052) | *82* | *H*(2) = 2.69, *p* = .261 | *82* |

*adjusted *p* for 18 tests <.003.
